# In Vitro Model of Metastasis to Bone Marrow Mediates Prostate Cancer Castration Resistant Growth through Paracrine and Extracellular Matrix Factors

**DOI:** 10.1371/journal.pone.0040372

**Published:** 2012-08-01

**Authors:** Reynald M. Lescarbeau, F. Philipp Seib, Marina Prewitz, Carsten Werner, David L. Kaplan

**Affiliations:** 1 Department of Biomedical Engineering, Tufts University, Medford, Massachusetts, United States of America; 2 Leibniz Institute for Polymer Research Dresden, Dresden, Germany; Henry Ford Health System, United States of America

## Abstract

The spread of prostate cancer cells to the bone marrow microenvironment and castration resistant growth are key steps in disease progression and significant sources of morbidity. However, the biological significance of mesenchymal stem cells (MSCs) and bone marrow derived extracellular matrix (BM-ECM) in this process is not fully understood. We therefore established an *in vitro* engineered bone marrow tissue model that incorporates hMSCs and BM-ECM to facilitate mechanistic studies of prostate cancer cell survival in androgen-depleted media in response to paracrine factors and BM-ECM. hMSC-derived paracrine factors increased LNCaP cell survival, which was in part attributed to IGFR and IL6 signaling. In addition, BM-ECM increased LNCaP and MDA-PCa-2b cell survival in androgen-depleted conditions, and induced chemoresistance and morphological changes in LNCaPs. To determine the effect of BM-ECM on cell signaling, the phosphorylation status of 46 kinases was examined. Increases in the phosphorylation of MAPK pathway-related proteins as well as sustained Akt phosphorylation were observed in BM-ECM cultures when compared to cultures grown on plasma-treated polystyrene. Blocking MEK1/2 or the PI3K pathway led to a significant reduction in LNCaP survival when cultured on BM-ECM in androgen-depleted conditions. The clinical relevance of these observations was determined by analyzing Erk phosphorylation in human bone metastatic prostate cancer versus non-metastatic prostate cancer, and increased phosphorylation was seen in the metastatic samples. Here we describe an engineered bone marrow model that mimics many features observed in patients and provides a platform for mechanistic *in vitro* studies.

## Introduction

The bone marrow microenvironment provides many cues which enable survival and proliferation of prostate cancer cells. It is now well established that, in particular, osteoblast secreted factors enable androgen independent growth of metastasized prostate cancer cells [1], [2]. In addition, bone marrow-derived extracellular matrix (BM-ECM) is implicated in the progression of other cancers, including multiple myeloma, via the activation of pathways associated with survival [3].

Due to the significant tropism of prostate cancer for bone marrow, numerous *in vivo* models have been developed to study this interaction. These include xenograft mouse models where cancer cells are injected intratibially [4], humanized mouse models where human bone is placed subcutaneously and then seeded with prostate cancer cells, subcutaneous implantation of tissue engineered bone [5], and hollow fibers containing both prostate cancer and bone like cells [6]. These *in vivo* models, however, are low throughput and potentially suffer from xenogenic interactions. To overcome some of these limitations, and to permit improved mechanistic and high throughput screening studies, researchers are using *in vitro* cell culture models. Only recently has conventional plasma treated polystyrene (PTP) tissue culture ware been replaced by culture substrates that more closely mimic the *in vivo* tumor microenvironment. These have included models studying paracrine interactions and, recently, ECM interactions [7], [8], [9]. However, none of these studies have used an engineered bone marrow tissue model in combination with androgen depleted media to model castration resistant prostate cancer progression *in vitro.*

The growth and survival of prostate cancer cells in androgen depleted conditions has been attributed to a variety of mechanisms. Up-regulation of the androgen receptor allowing for increased sensitivity, mutations enabling increased promiscuity to other ligands, as well as intracrine synthesis of androgen by the prostate cancer cells contribute to androgen independent growth [10]. Other methods of androgen independent growth include the activation of mitogenic pathways and transcription factors such as MAPK, PI3K, STAT3, and β-Catenin [11], [12]. Activation of these pathways enables androgen independent growth or cell survival through co-activation of the androgen receptor.

Previous *in vitro* studies suggested that single ECM components may act as ligands to induce mitogenic cell signaling and survival. For example, fibronectin, which is a component of BM-ECM, induced MEK phosphorylation through FAK and Src [13]. Similarly, integrin β1 and the IGF-1R interactions with fibronectin mediated chemoresistance in the prostate cancer cell line DU145 [14]. These studies indicate a possible role for BM-ECM ligands activating pathways associated with androgen independent growth and disease progression.

**Figure 1:**
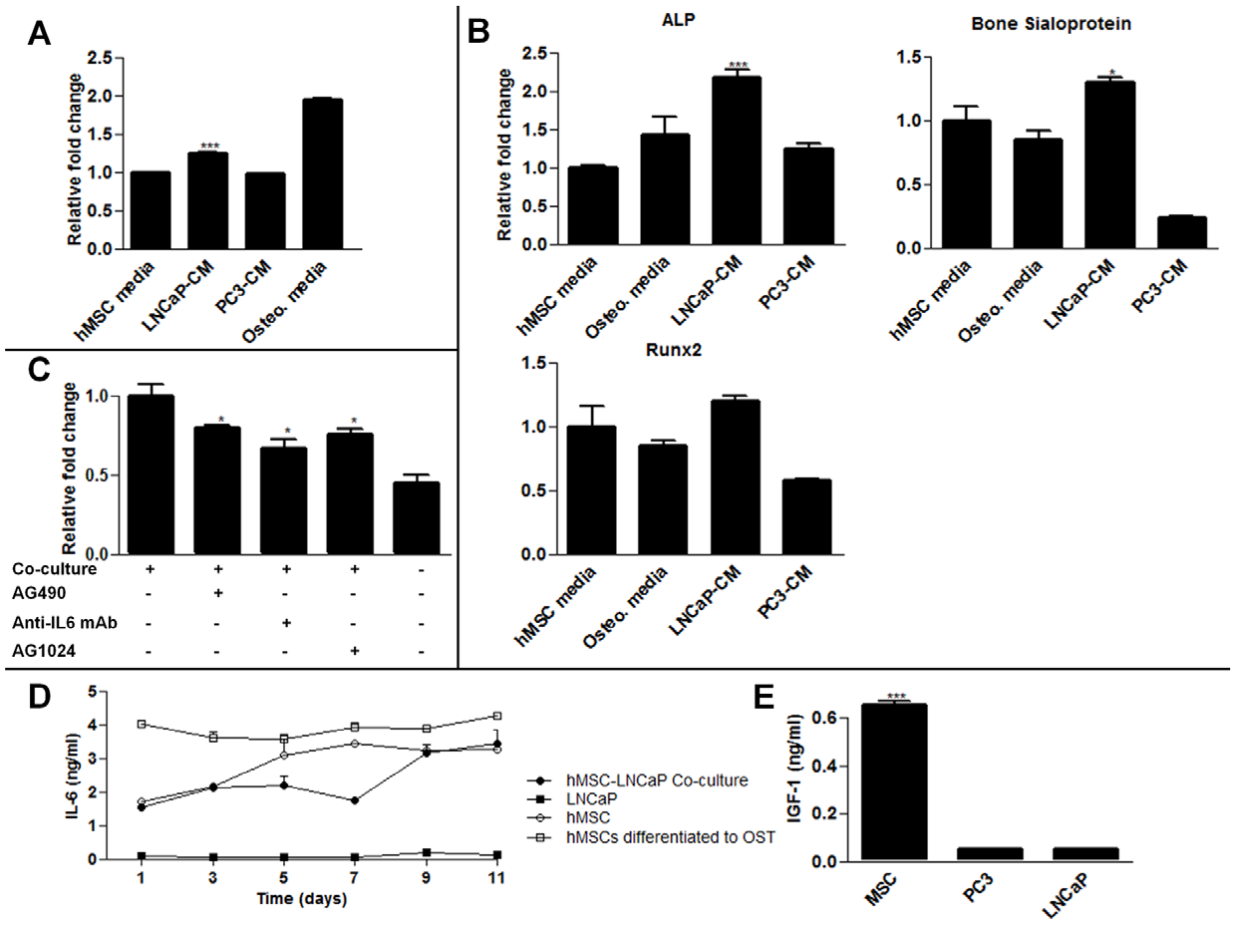
hMSC-LNCaP paracrine signaling. Co-cultures of human mesenchymal stem cells (hMSCs) and prostate cancer cells permits paracrine signaling, induces osteogenesis, and supports prostate cancer survival. For panels A, B, C raw values were normalized to the negative control group. (A) hMSCs were treated with normal growth media, LNCaP conditioned media, PC3 conditioned media, or osteogenic media 14 days. They were then stained with alizarin red which was subsequently extracted and quantified to determine calcium deposition. (B) qRT-PCR for the specified transcripts. Each group was cultured as described in (A), and RNA subsequently extracted. (C) Indirect co-cultures of hMSCs with LNCaPs in androgen deleted media in the presence of the IGFR inhibitor AG1024, or IL6 inhibitors, AG490 and anti-IL6 mAb. Cells were plated in growth medium, allowed to recover for 24 h, and subsequently switched to androgen depleted medium plus inhibitor. LNCaP viability was measured after 7 days. (D) IL6 concentrations in conditioned culture medium. (E) IGF-1 concentrations in conditioned media after 48 hours from each cell type cultured alone. (*P<0.05; **P<0.01; ***P<0.001, n = 3, ± SEM).

**Figure 2:**
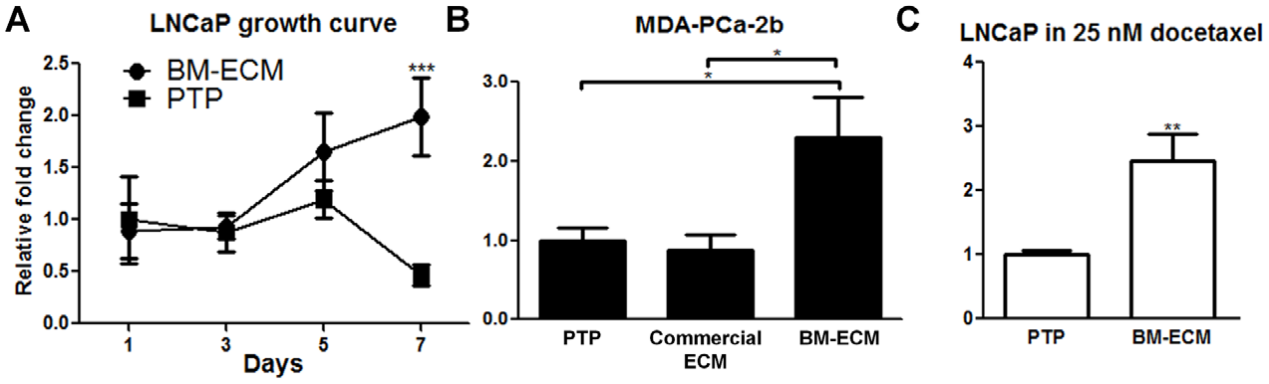
Effect of bone marrow derived extracellular matrix (BM-ECM) on prostate cancer cells. Bone marrow-derived extracellular matrix (BM-ECM) protects prostate cancer cells against androgen depletion and chemotherapy. For panels A, B, C raw values were normalized to the negative control group. (A) Growth of LNCaP cells over time cultured in androgen depleted media as measured via MTT. Equal numbers of LNCaPs were seeded to BM-ECM or PTP with an n of 4 per group per time point, and assayed on the appropriate day. (B) MDA-PCa-2bs were plated and switched to androgen deleted media as described in (A) and cell viability was determined after 4 days. MDA-PCa-2bs cells were also cultured on commercial ECM. (C) Cell viability of LNCaP cells after 72 hours in growth medium with 25 nM docetaxel. (*P<0.05; **P<0.01; ***P<0.001, n = 3, except as noted in (A), ± SEM).

**Figure 3:**
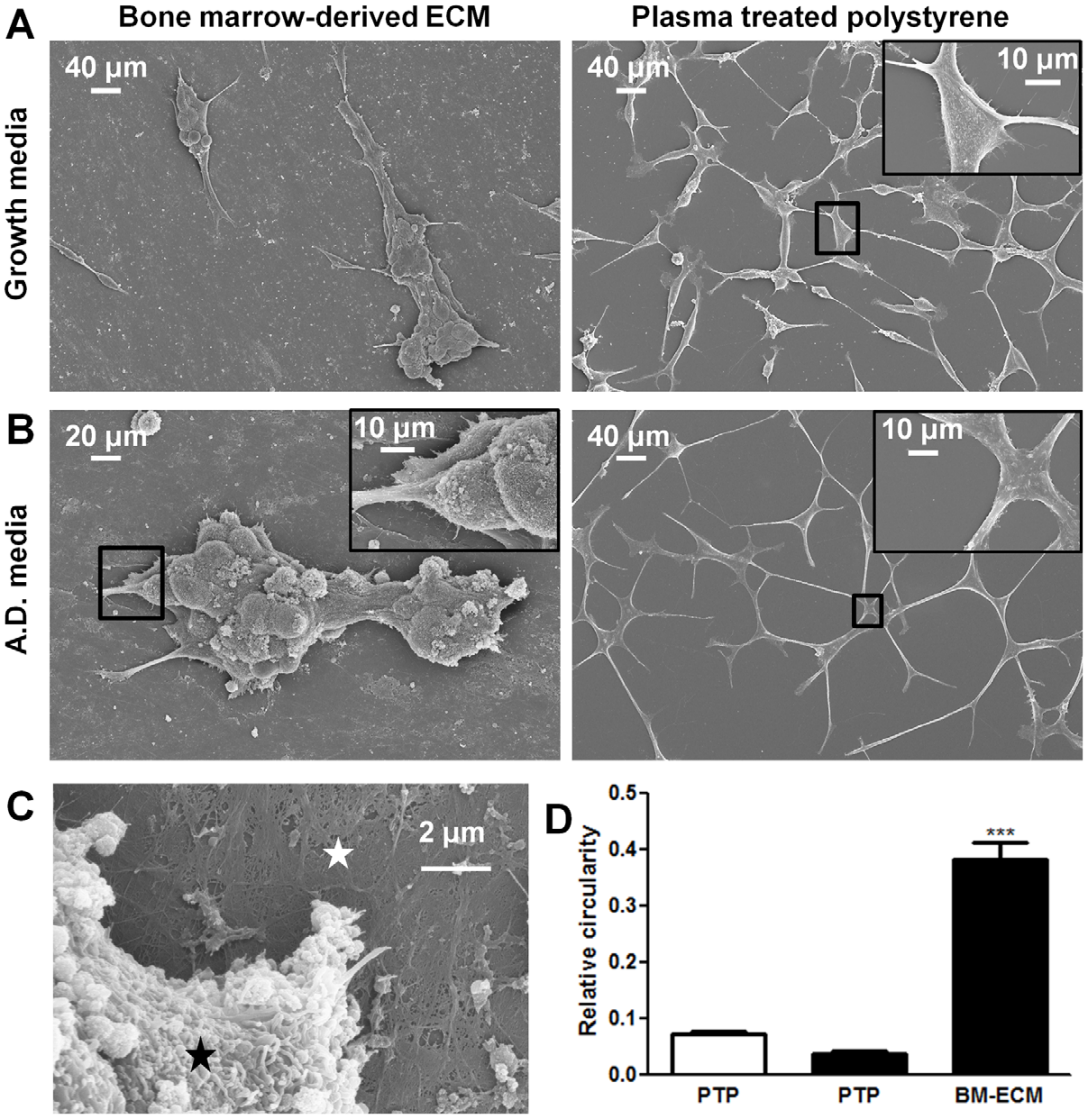
Scanning electron micrographs of LNCaP cells. Bone marrow-derived extracellular matrix (BM-ECM) induces cluster like growth of LNCaPs that resists androgen depletion. LNCaPs were seeded to BM-ECM and PTP as detailed in Figure 2A. Scanning electron micrographs of 7 day cultures in (A) normal growth media and (B) androgen depleted media. (C) Higher magnification scanning electron micrograph of LNCaP (black asterisk) cells cultured on BM-ECM (white asterisk). (D) The circularity of LNCaPs on PTP in androgen depleted and growth medium and LNCaPs on BM-ECM in androgen depleted media was quantified from scanning electron microscopy images after 7 days in culture (*P<0.05; **P<0.01; ***P<0.001, ± SEM).

**Figure 4:**
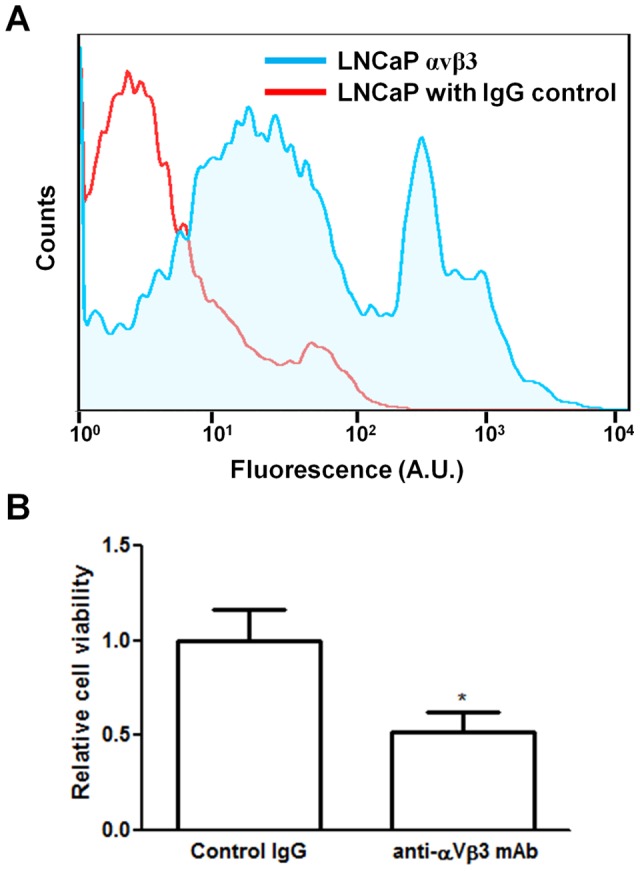
Integrin αvβ3 mediated adhesion to BM-ECM. Expression of αvβ3 integrin on LNCaP cells mediates adhesion to bone marrow-derived extracellular matrix (BM-ECM). (A) Cell surface expression of αvβ3 integrin as measured by flow cytometry. (B) Anti-αvβ3 integrin antibody reduced cell adhesion to BM-ECM. Raw values were normalized to the control IgG group. (n = 3, *P<0.05; **P<0.01; ***P<0.001, ± SEM).

**Figure 5:**
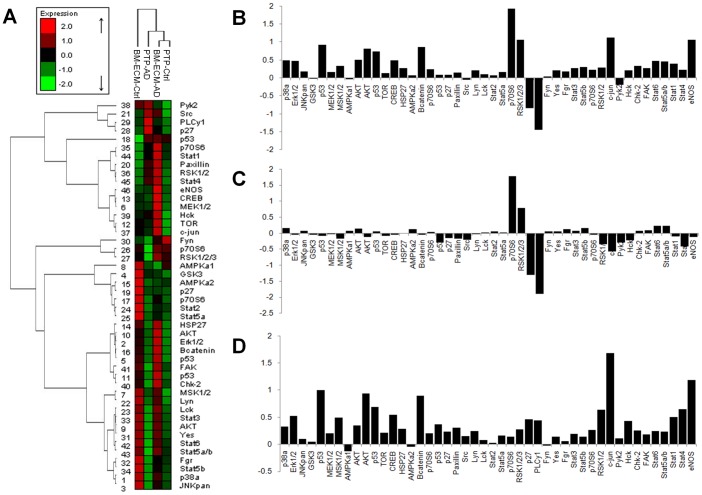
LNCaP phosphoproteomic analysis. Differential phospho-proteomics of LNCaP cells cultured on bone marrow-derived extracellular matrix (BM-ECM). (A) Hierarchical clustering reveals LNCaPs on BM-ECM in androgen depleted media clustered most closely with LNCaPs cultured on PTP in growth media (B) Differential phosphorylation of LNCaPs on BM-ECM in androgen depleted media versus LNCaPs on PTP in androgen depleted media. (C) Differential phosphorylation of LNCaPs on PTP in androgen depleted media versus LNCaPs on PTP in growth media. (D) Differential phosphorylation of LNCaPs on BM-ECM in androgen depleted media versus LNCaPs on PTP in growth media.

**Figure 6:**
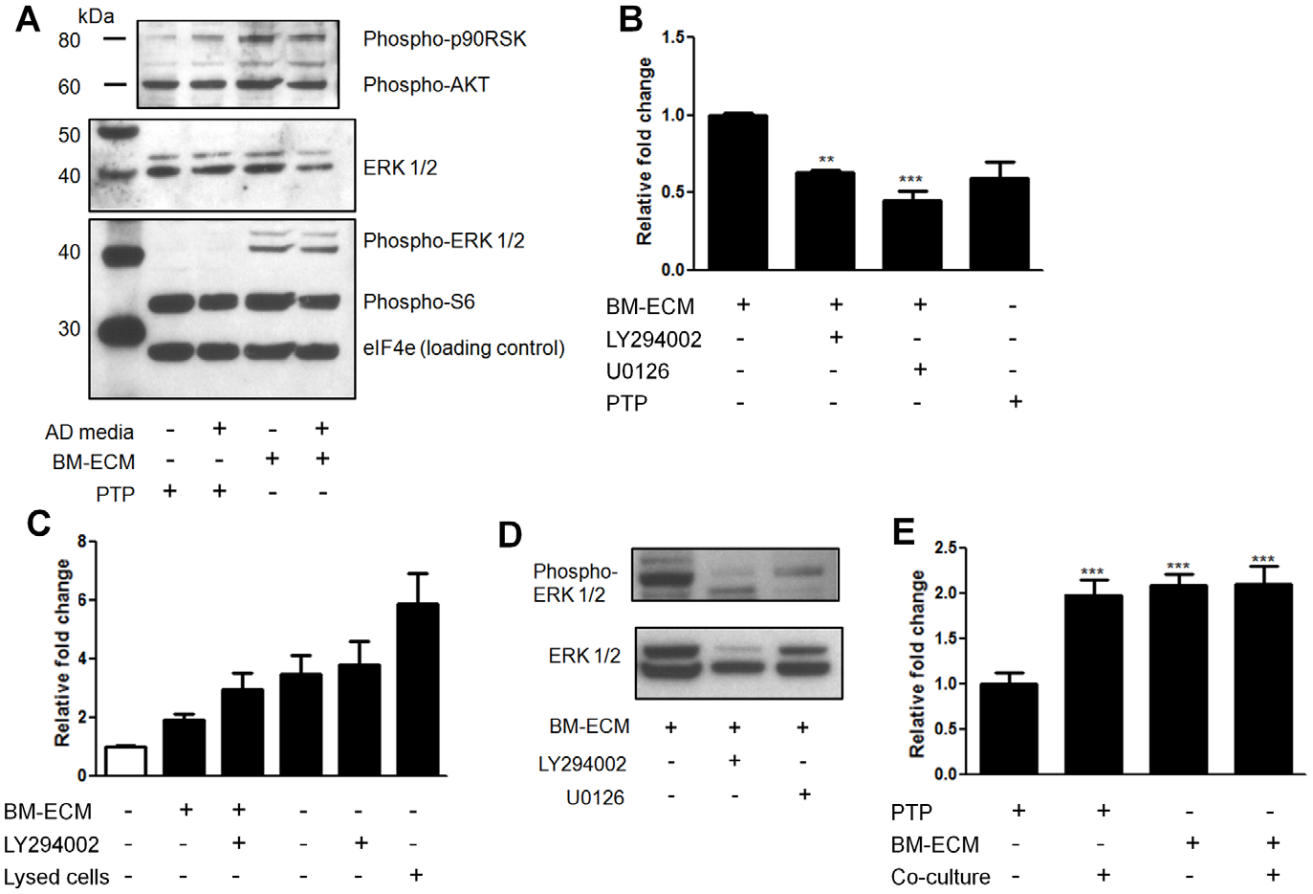
Effect of inhibitors on LNCaP pathway activation and survival on BM-ECM. Bone marrow-derived extracellular matrix (BM-ECM) induces differential signaling in LNCaP cells that mediates cell survival. Figure panels C, D, E raw values were normalized to the negative control group. (A) Western blot of LNCaPs indicates increased phospho-Erk1/2 and phospho-p90RSK in response to BM-ECM. (B) Effect of PI3K, LY294002, or MEK1/2, U0126, inhibitors on LNCaPs cultured on BM-ECM. Cells were plated at equal densities to BM-ECM or PTP in growth media and after 24 h switched to androgen depleted media ± inhibitors. Cell viability was measured after 7 days (n = 3). (C) Relative apoptosis as measured using a glucose 6-phosphate dehydrogenase assay after 72 hours. Clear bar indicates growth media, black bars androgen depleted media. Lysed cells are a positive control (n = 8). (D) LNCaPs were cultured on PTP indirectly with hMSCs, on BM-ECM, or in both conditions simultaneously while in androgen depleted media. Relative cell viability was measured after 7 days (n = 3, except as noted in (D), *P<0.05; **P<0.01; ***P<0.001, ± SEM). (E) Reductions in phospho-Erk1/2 in LNCaP cells on BM-ECM when treated with U0126 and LY294002.

**Figure 7:**
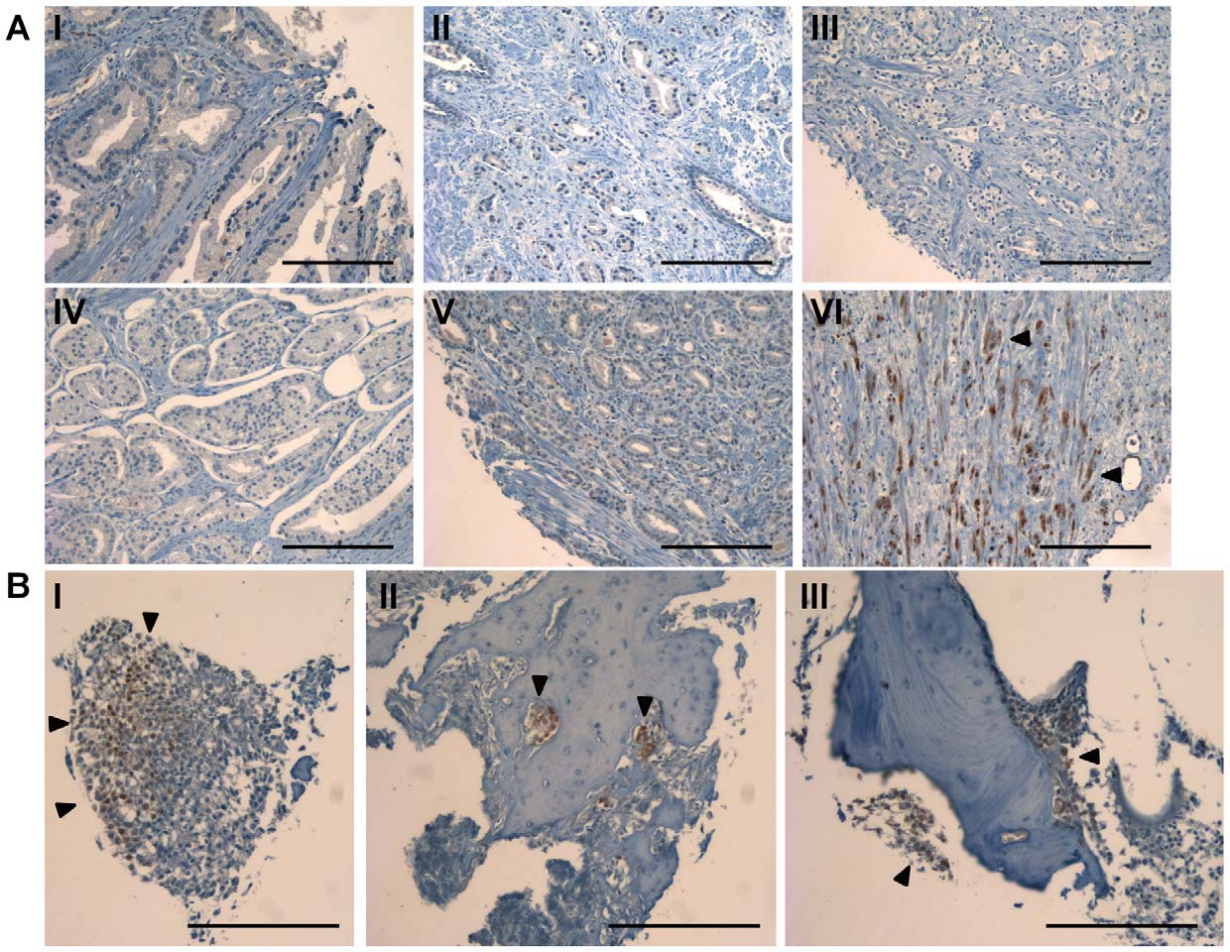
Phospho-Erk immunohistochemistry in human prostate tissue sections. Staining for Erk phosphorylation on a tissue microarray from primary tumor samples, and bone metastatic samples (A) Representative tissue sections stained for p-Erk from primary prostate tumor samples. Sample VI contained the only substantial positive staining in primary prostate samples. (B) Tissue sections stained for p-Erk from bone metastatic prostate cancer samples (scale bars are 200 µm).

**Figure 8:**
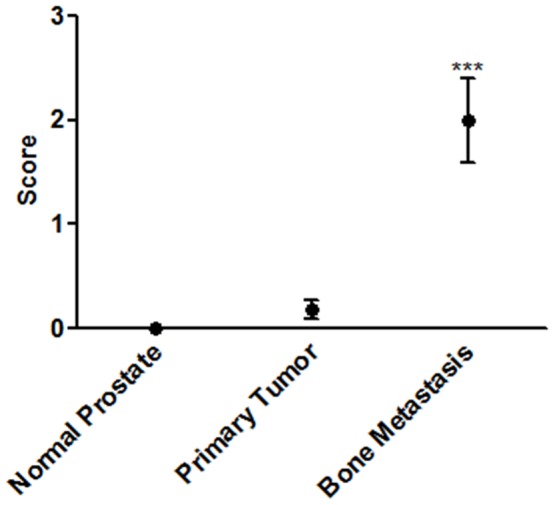
Quantification of phospho-Erk immunohistochemistry. The mean score plotted for each group taken from two blinded investigators (black circles). Staining in bone metastasis sections is significant over both normal tissue and primary tumor sections (n = 8 for normal prostate tissue, n = 72 for primary tumor, and n = 4 bone metastasis, ***P<0.001, ± std. dev.).

Here we present an *in vitro* platform, which enables efficient and accurate examination of the bone marrow tumor microenvironment with a specific focus on the BM-ECMs. We used this system to examine the response of prostate cancer cells and were able to indentify numerous factors that contributed to disease progression including androgen independent growth and chemoresistance. The identified factors included IGF1 and IL6 paracrine signaling, and activation of the MAPK pathway via BM-ECM signaling.

## Materials and Methods

### Cell culture and BM-ECM substrate

LNCaP, PC3, and MDA-PCa-2b cells were recently purchased from ATCC (Manassas, VA) which validates cell lines using STR analysis. Whole bone marrow aspirates were obtained from Lonza (Basel, Switzerland), and hMSCs were isolated and characterized as detailed elsewhere [15]. After isolation, hMSCs were cultured in DMEM supplemented with 10% fetal bovine serum (FBS), 1% Penicillin Streptomycin, 0.1 mM nonessential amino acids, and 1 ng/ml basic fibroblast growth factor. LNCaP and PC3 cells were grown in RPMI 1640 supplemented with 10% FBS, and 1% Penicillin Streptomycin. While MDA-PCa-2b cells were were grown in BRFF-HPC1 medium (Athena ES, Baltimore, MD) supplemented with 20% FBS with no Penicillin Streptomycin. For studies involving androgen depleted media, phenol red free base media was used with 10% charcoal stripped FBS. Indirect co-cultures used 1 µm pore size transwell inserts (BD Biosciences, Franklin Lakes, NJ) with one cell type seeded on the tissue culture plate and another on the cell culture insert. Indirect co-culture studies used a 1∶1 ratio of media from the two cell types of interest. In androgen depleted studies both media types were prepared as described above. Cell cultures were maintained at 37°C in a humidified atmosphere of 5% CO_2_. The bone marrow-derived ECM was generated as detailed elsewhere [16]. Unless otherwise noted all cell culture reagents were acquired from Invitrogen (Carlsbad, CA).

### Androgen depleted growth studies

For androgen independent growth/survival studies, prostate cancer cells were seeded at 5,000 cells/cm^2^ in growth media on either PTP or BM-ECM. Studies including MDA-PCa-2b cells, PTP control plates were also coated first with a proprietary cell attachment matrix that contains fibronectin, collagen and albumin (Athena ES, Baltimore, MD) to facilitate cell adhesion. Cells were allowed to adhere for 24 hours, after which the cultures were washed with PBS, and switched to androgen depleted media. LNCaP cells on BM-ECM or PTP were assayed as described below at their respective time points to complete the growth curve, or the media was replaced. MDA-PCa-2b cells were culture for 4 days, media was changed at 2 days, and the relative cell numbers assessed via 3-(4,5-dimethylthiazol-2-yl)-2,5-diphenyltetrazolium bromide (MTT, Invitrogen) at the end of the culture period following the manufacture's protocol. The validity of the MTT assay was confirmed by comparing to manual counting and DNA quantification (Fig. S1A, S1B). MTT values were normalized to the negative control which was arbitrarily set to a value of one.

### Integrin blocking experiment

Suspended LNCaP cells were pre-incubated with a blocking monoclonal antibody against αvβ3 (20 µg/ml), clone 23C6 (eBioscience, San Diego, CA), or IgG isotype control for 30 minutes and then seeded to BM-ECM. After 12 hours, the media was aspirated and the cells were washed once with PBS. The relative cell number was then measured using a MTT assay as described above.

### Docetaxel treatment studies

Prostate cancer cells were seeded on PTP or BM-ECM as detailed above and allowed to recover for 24 hours. Next, cells were washed with PBS and replaced with growth medium supplemented with 25 nM docetaxel or vehicle only. Cells were exposed to treatment for 72 hours and the relative cell viability was then measured using a MTT assay, which was normalized as in previous MTT assays.

### ELISA assays

To collect conditioned media samples each cell type was seeded to PTP and co-cultures conducted using transwell inserts with hMSCs on PTP and LNCaP cells seeded to the inserts. Conditioned media from each group was removed every 48 hours and replaced with fresh media. Both IL6 and IGF-1 ELISA kits were performed according to manufacturer's instructions (eBioscience and R&D Systems, respectively). The experiment was run in triplicate and the mean value calculated.

### Apoptosis assay

LNCaPs were seeded at 2,500 cells/cm^2^ in a 24 well plate and allowed to adhere for 24 hours before the media was changed to either growth media, androgen depleted media, or androgen depleted media supplemented with LY294002 (EMD Chemicals, Gibbstown, NJ). After 72 hours media was collected from each well and assayed for apoptosis using a glucose 6-phosphate assay (Invitrogen) according to manufactures instructions. 0.25% Triton X-100 was supplemented in the positive control group prior to the removal of the media. The experiment was run with n = 8 and the mean calculated. Measured fluorescence values were normalized to the negative control which was set to a value of one.

### Scanning electron microscopy

LNCaP cells were seeded on PTP or BM-ECM as detailed above. At 4 days in growth- or androgen depleted media, cultures were terminated, washed with PBS, fixed with 2% glutaraldehyde and prepared for platinum-palladium sputtering as detailed elsewhere [17]. Samples were visualized with a Zeiss Supra 55VP field emission scanning electron microscope.

### Western blot and reverse phase protein arrays

LNCaP cells were seeded in growth medium to PTP and BM-ECM and allowed to recover for 24 hours. As indicated, culture medium was either replaced with fresh growth medium or androgen depleted media and cells were cultured for 72 hours. Next, media was removed, cells were washed with ice cold PBS and harvested with a cell scraper. Cells were pelleted at 4°C and lysed with RIPA buffer (Roche, Basel, Switzerland) that contained protease and phosphatase inhibitors (Pierce Biotechnology, Rockford, IL) cocktails at 4°C The protein content was determined with the bicinchoninic acid protein assay (Pierce Biotechnology) and 5 µg of cellular protein was reduced and analyzed by Western blotting per lane. Membranes were blocked with bovine serum albumin and probed with a cocktail of antibodies against phospho-p90RSK (Ser380), phospho-Akt (Ser473), phospho-p44/42 (Thr202/Tyr204), phospho-S6 (Ser235/236), phospho-Erk1/2 (Thr202/Tyr204), and eIF4E (Cell Signaling Technology, Danvers, MA) using an overnight incubation at 4°C and a 1∶400 antibody dilution. Total ERK was determined using a p44/42 MAPK specific antibody (Cell Signaling Technology) at a 1∶2000 dilution. For the reverse phase protein arrays, at total of 150 µg of LNCaP lysate was assayed using the human phospho-kinase proteome profiler kit (R&D Systems, Minneapolis, MN) according to manufacturer's instructions.

### Flow cytometry

LNCaP cells were trypsinized and spun down into a pellet (1,200 RPM, 10 minutes). The cells were then resuspended in a PBS containing the αvβ3 antibody, LM609 clone, (Millipore, Billerica, MA) at a dilution of 1∶300 and incubated at 4°C for 30 min. The cells were then fixed in 4% formalin, and analyzed using flow cytometry (FACSCaliber, BD Biosciences). The data was analyzed using the Flow-jo software (Tree-star Inc., Ashland, OR).

### Alazarin red stain and quantification

To assess osteogenic differentiation of hMSCs in response to prostate cancer cell conditioned media, LNCaPs and hMSCs or PC3s and hMSCs were indirectly co-cultured in a 1∶1 ratio of the respective media. At 14 days of co-culture, the MSC layer was fixed with 4% formalin and incubated with an aqueous 1% alizarin red solution (pH 4.2) (Sigma-Aldrich) for 10 min at room temperature. Next, samples were washed with water to remove unbound dye and subsequently imaged by light microscopy. For alizarin red quantification 10% acetic acid was added to each well, incubated for 30 minutes, removed from each well, and neutralized with 10% ammonium hydroxide. Finally, the absorbance of the samples was read at 405 nm using a spectrophotometer. Absorbance values were normalized to the negative control which was set to a value of one.

### Signaling inhibition studies

For indirect co-cultures cells were seeded in growth media and after 24 h the media was replaced with a 1∶1 mixture of LNCaP and hMSC androgen depleted media. This media was supplemented with LY294002 at a concentration of 5 µM or U0126 (EMD Chemicals) at 100 nM. AG1024 and AG490 (EMD Chemicals) were also supplemented into the androgen depleted media of hMSCs and LNCaPs in indirect co-culture at a concentration of 1 µM and 50 µM, respectively. The anti-human IL6 monoclonal antibody (eBioscience) was supplemented into the media at a concentration of 20 µg/ml while LNCaPs were indirectly co-cultured with hMSCs. Working concentrations of small molecule inhibitors were determined from dose response curves of LNCaPs alone in culture (Fig. S2). Cell survival was determined with the MTT assay as detailed above after 7 days. MTT values were normalized to the negative control which was set to a value of one.

### Quantitative real time polymerase chain reaction (RT-PCR)

Total RNA was isolated using RNeasy silica columns (Qiagen, Valencia, CA) according to the manufacturer's instructions, and 200 ng of RNA was reverse transcribed from each sample (iScript, Bio-Rad, Hercules CA). The expression levels of target genes were quantified by RT-PCR using Brilliant II Fast QPCR master mix (Aglient Technologies, Santa Clara CA) and validated primers (TaqMan, Applied Biosystems, Carlsbad CA). The relative expression of target genes was calculated based on the C_T_ values, and these were normalized to that of the housekeeping gene glyceraldehyde 3-phosphate dehydrogenase using the 2^−ΔΔCT^ method assuming 100% PCR efficiency [18].

### Phospho-Erk immunohistochemistry

Prostate tissue microarrays containing sections of normal prostate tissue, primary prostate tumors, and bone metastatic prostate tissue were obtained from US Biomax (Rockville, MD). A monoclonal antibody against the Erk1/2 phosphosites T185, Y187, T202, and Y204 was obtained from Abcam (Cambridge, MA), clone MAPK-YT. Staining was performed by the Tufts Medical Center Histology laboratory following optimization of antibody dilution and blocking on optimizations slides cut from the same tissue blocks as the experimental samples. Brightfield microscopy was performed using a Leica Microsystems DM IL microscope (Buffalo Grove, IL), with a Leica Microsystems DFC420 camera, and Leica Microsystems Application Suite V3.5 software with no alterations to raw images. Sections were scored by two blinded investigators using the Dako scale (0,1+,2+,3+) where final scores for each section were then averaged together [19]. A one way ANOVA test was used with Bonferroni's post hoc test at the 95% confidence interval to determine significance.

### IC50 calculations

LNCaP cells were seeded in a 96 well plate at 18,000 cells/cm^2^. The cells were treated with increasing concentrations of inhibitor and relative survival measured with a MTT assay after 3 days. IC50 concentrations were determined based on half the distance between the maximum and minimum survival values along the dose response curve. The mean was taken of 12 replicates per concentration.

### Validation of cell count

Equal numbers of LNCaP cells were plated to PTP and BM-ECM in a 24 well plate at 2,500 cells/cm^2^, 5,000 cells/cm^2^, and 7,500 cells/cm^2^, as determined via counting with a hemocytometer. Additionally, increasing concentrations of LNCaP cells were prepared and a measurement taken from each sample for MTT (Invitrogen, Carlsbad, CA) and DNA quantification assays using Picogreen (Invitrogen, Carlsbad, CA) to create a standard curve. After 24 hours of culture in androgen depleted media, cells on PTP and BM-ECM were assayed using DNA quantification, MTT, and manual counting via nuclear staining and imaging. Cell nuclei were stained with Hoechst 33258, washed with PBS, and imaged at 20x using a Leica DM IL inverted microscope. Five representative images were taken per well and the number of nuclei manually counted. This number of nuclei was averaged and multiplied by the surface area of a single well of a 24 well plate (2 cm^2^) over the area of the field of view at 20x to determine the number of cells per well, and the average of all biological replicates taken (n = 4).

### Data analysis

Statistical analysis was completed using Graphpad Prism 5. Significance was accessed using unpaired one tailed t-tests at the 95% confidence interval, or one way ANOVA with Dunnett's multiple comparison test, also at the 95% confidence interval, unless otherwise noted (*P<0.05; **P<0.01; *** P<0.001). Data was represented as the mean ± SEM, unless otherwise noted. Image circularity analysis was completed using ImageJ and suggested thresholding settings. Increasing values on the circularity scale indicate increasingly circular cells. Protein microarray data were scanned as an image file and the integrated pixel intensity for each point measured using ImageJ. The average of duplicates was taken, the data was mean centered, and hierarchical clustering analysis was performed. Differential protein phosphorylation between groups was expressed as the difference of two measurements divided by the population standard deviation.

## Results

### Paracrine interactions of hMSCs and LNCaPs promote osteogenic differentiation of hMSCs and androgen independent survival of LNCaPs

To better understand the paracrine signaling between hMSCs and prostate cancer cells, we used indirect co-cultures of LNCaPs and hMSCs. LNCaPs induce osteogenic differentiation of hMSCs (Fig. 1A, 1B) which resembled osteoblast activation frequently seen in prostate cancer patients [20]. Additionally, the survival of LNCaP cells in hMSC co-cultures was significantly enhanced over monotypic LNCaP cultures in androgen depleted media (Fig. 1C). To interrogate these effects, IL6 signaling was inhibited with an anti-IL6 monoclonal antibody or, AG490, a small molecule inhibitor of the Jak2 kinase, an upstream activator of Stat3. These treatments significantly reduced the viability of the LNCaPs compared to the uninhibited co-culture group in androgen depleted conditions (Fig. 1C). Noting that the reduction in viability was not to the level of LNCaPs cultured alone, we also tested the effect of AG1024, an IGFR inhibitor. This drug also significantly reduced viability of the LNCaP cells indirectly co-cultured with hMSCs. These inhibitors alone were not cytotoxic to the cells (Fig. S2). Finally, the expression of IL6 over time was confirmed via ELISA from hMSCs conditioned media with different conditions (Fig. 1D). Notably, hMSCs which had been pre-differentiated towards an osteogenic linage consistently had higher rates of IL6 expression. Considering LNCaPs induce hMSCs to undergo osteogenic differentiation, this may be a source of increase IL6 ligands levels. Additionally, IGF-1 levels were measured in conditioned media with an ELISA assay. No time varying effects were seen, however significant steady state release of hMSCs was observed as compared to PC3 and LNCaP cells (Fig. 1E).

### BM-ECM promotes survival of LNCaP cells in androgen depleted media and chemoresistance

In addition to soluble paracrine signals, we hypothesized that bone marrow-like ECM may play a role in the survival of androgen-dependent prostate cancer cells. A decellularized matrix was derived from ECM proteins secreted by hMSCs [16]. After one week of culture in androgen deprived media there was a significant increase in the number of viable cells on BM-ECM as compared to cells in androgen depleted media on PTP (Fig. 2A). The mechanism of this effect was confirmed on a second androgen-dependent cell line, namely MDA-PCa-2b cells (Fig. 2B). MDA-PCa-2b cells are routinely cultured on commercially available ECM to support cell adhesion and growth. However, this ECM substrate did not support the growth of MDA-PCa-2b cells in androgen depleted conditions indicating the effect is specific to BM-ECM components or structure (Fig. 2B). Next, we examined whether BM-ECM could confer chemoresistance using growth medium supplemented with docetaxel. BM-ECM cultured LNCaP cells showed a significant increase in cell survival when compared to PTP cultures (Fig. 2C). These data indicate that the components of the BM-ECM play a role in determining survival in response to androgen deprivation and docetaxel treatment.

### BM-ECM alters LNCaP morphology

To provide a better understanding of the BM-ECM and LNCaP crosstalk, we analyzed cell morphology by scanning electron microscopy. LNCaPs could be observed attached to ECM fibers and matrix (Fig. 3A, 3B, and 3C). LNCaP cells cultured in androgen depleted media had a substantial increase in elongation as quantified following SEM imaging after 4 days (Fig. 3B). However, this morphological change was prevented from occurring when the cells were cultured in androgen depleted media on BM-ECM. In this condition the cells appeared to grow in clusters resulting in a significant increase in measured circularity (Fig. 3D).

### Blocking αvβ3 integrin limits attachment to BM-ECM

Next, we sought to examine the role of the αvβ3 integrin complex for BM-ECM attachment, because this integrin complex has been implicated in ECM attachment and subsequent bone marrow metastasis [21]. Flow cytometry showed cell surface labeling for αvβ3 integrin (Fig. 4A). To determine functional significance, suspended cells were incubated with a blocking antibody against αvβ3 integrin or isotype control and cell attachment to BM-ECM was then measured. A significant decrease in cell attachment was measured, suggesting that the αvβ3 integrin in this case plays a role in mediating attachment to BM-ECM (Fig. 4B).

### Effect of BM-ECM on MAPK and PI3K signaling pathways

To determine the mechanism mediating the response of the LNCaPs to BM-ECM we examined the relative phosphorylation state of 46 proteins from multiple pathways using a reverse phase protein microarray. When hierarchical clustering analysis was performed, LNCaPs on BM-ECM in androgen depleted media clustered most closely with LNCaPs in growth media on PTP (Fig. 5A). Differential phosphorylation was then assessed between LNCaPs on BM-ECM in androgen depleted media and LNCaPs on PTP in androgen depleted media, and numerous proteins appeared to be differentially phosphorylated (Fig. 5B). However, when we also examined phosphorylation differences between LNCaPs on PTP in growth media versus androgen depleted media several proteins were similarly regulated (Fig. 5C). These alterations appeared to be specific to LNCaPs cultured on PTP in androgen depleted media, and may be related to growth arrest. The closely clustered groups of LNCaPs on BM-ECM in androgen depleted media and LNCaPs on PTP in growth media were examined (Fig. 5D). Increases in phosphorylation of numerous proteins involved in MAPK signaling was observed, including MEK1/2, Erk1/2, and c-jun, as well as moderate increases in Akt. A Western blot was used to confirm these alterations in signaling, which indicated substantial increases in Erk1/2 phosphorylation (Thr202/Tyr204), and sustained but not increased phospho-Akt (Fig. 6A).

### Blocking MAPK and PI3K pathways inhibit BM-ECM mediated androgen independent survival

Following the observation that Erk1/2 had consistently increased phosphorylation we determined the effect of blocking this pathway on the BM-ECM mediated cell survival. LNCaP cells were cultured in androgen depleted media containing U0126, a MEK inhibitor. A significant decrease in cell survival was observed in the treated cells, compared to cells culture on BM-ECM in androgen depleted media (Fig. 6B). This reduction was to the level of LNCaPs cultured on PTP in androgen depleted media. We additionally sought to determine the role of PI3K signaling in enabling androgen independent growth. Consistent with previous reports on PTP, treating LNCaPs on BM-ECM in androgen depleted conditions with LY294002, a PI3K inhibitor, resulted in substantial apoptosis (Fig. 6C). In contrast, LY294002 treatment in growth media did not result in any significant changes in proliferation (data not shown). Both U0126 and LY294002 were observed to reduce the level of p-Erk1/2 in LNCaP cells on BM-ECM when preincubated with the cells (Fig. 6D, Fig. S3). The effects of both BM-ECM and paracrine signals were also examined through combination culture of LNCaPs on BM-ECM with simultaneous indirect co-culture with hMSCs. There was no measured increase in relative cell viability over LNCaPs indirectly co-cultured with hMSCs or LNCaPs on BM-ECM (Fig. 6E), necessitating further studies to determine the dynamics of these signaling mechanisms.

### Immunohistochemistry for p-Erk in human primary and metastatic prostate tumor

To determine whether phospho-Erk may be activated in human prostate cancer we performed immunohistochemistry on a tissue microarray containing samples from normal prostate tissue, primary prostate tumors of various stages, and bone metastatic prostate cancer. Examination of the normal prostate and primary tumor indicated minimal p-Erk staining present (Fig. 7A, 8). Only one section was observed to have substantial staining in primary tumor sections (Fig. 7A, bottom right). However, bone metastatic samples had significantly increased p-Erk staining as compared to non-metastatic samples when scored and quantified (Fig. 7B, 8).

## Discussion

In patients with prostate cancer a major source of morbidity is due to widespread metastases to the bone marrow, which often grow in a castration resistant manner. Androgen independent growth makes prostate cancer an incurable disease due to the lack of effective drug therapies that target metastasized prostate cancer cells. Monotypic *in vitro* cultures on conventional PTP are commonly employed for initial screening studies to identify potential drug candidates for clinical development. However, such screening methods fail to identify drugs targeting pathways activated by stromal interactions. Therefore there is the need to use systems that more closely recapitulate the *in vivo* tumor microenvironment. We established an *in vitro* bone marrow model that provides the tumor cells with contextual signals and permits studies into microenvironment mediated signaling. Although this model is a 2D cell culture system, significant alterations consistent with malignant progression are observed.

We observed osteogenesis and increased LNCaP survival during hMSC co-cultures, which closely resembled the osteoblastic nature of prostate cancer metastasis in humans. These effects were partially abrogated when the co-cultures were treated with IL6 and IGFR inhibitors. The downstream effectors of IL6 and IGF signaling, Stat3 and the PI3K pathway have both been implicated in promoting androgen independent progression [7], [22]. The highest IL6 levels were observed for hMSCs differentiated into osteoblasts and thus may represent a positive feedback loop between prostate cancer cells and hMSCs.

In addition to paracrine factors, we also examined the importance of cell attachment to BM-ECM for androgen independent growth of prostate cancer cells. Although the exact BM-ECM composition mediating this effect remains undefined, reports have indicated similar ECMs contain collagen type I, fibronectin, osteopontin, as well as numerous growth factors embedded directly in the matrix [8]. Previous studies showed that fibronectin increased Erk1/2 phosphorylation, and prostate cancer cells contained in a 3D ECM of Matrigel were more resistant to treatment than those cultured in 2D [14], [23]. Importantly, a commercially obtained ECM containing collagen, albumin, and fibronectin for the culture of MDA-PCa-2b cells did not induce androgen independent growth in these cells (Fig. 2B). This suggests that a unique composition of BM-ECM is critical to evoke androgen independent growth and survival.

The BM-ECM also enabled chemoresistance in LNCaP cells when treated with docetaxel when compared to cells on PTP. Interestingly, there have been varying reports on the effectiveness of MAPK activation causing chemoresistance in prostate cancer cells. Unlike other cancer types, studies have indicated that MAPK activation may not increase chemoresistance [24]. There may be additional pathways activated due to BM-ECM attachment which are mediating chemoresistance [14].

In addition to androgen independent growth and chemoresistance, the BM-ECM mediated changes in morphology of LNCaP cells when compared to the cells on PTP. It has been well established that LNCaP cells cultured on PTP in androgen depleted media undergo apoptosis and neuroendocrine differentiation [25]. EGF treatment prevented neuroendocrine differentiation in these conditions, through activation of the MAPK pathway [26]. The similarity to the conditions presented here, where the cells cultured on BM-ECM had substantially increased ERK1/2 phosphorylation, may suggest a mechanism for this morphological difference.

When the mechanism of LNCaP attachment to BM-ECM was investigated, we demonstrated that attachment to this ECM was at least partially mediated through the αvβ3 integrin complex. Previously, there have been conflicting reports on the expression of this integrin on LNCaP cells. Our flow cytometry data indicated robust expression, and a functional role in BM-ECM attachment. Further studies are needed to specifically elucidate how this integrin, in conjunction with BM-ECM derived ligands may be signaling the MAPK pathway.

The phosphorylation state of 46 kinases was examined via reverse phase protein array and a consistent increase in the activation of MAPK related proteins was observed in LNCaP cells on BM-ECM (Fig. 5A), which was confirmed with a phospho-Erk1/2 western blot (Fig. 6A). The pro-survival effect of the BM-ECM was largely abrogated when the LNCaP cells were treated with a MEK inhibitor, further validating the MAPK pathway as a mechanism driving androgen independent growth. LNCaP cells are also known to have lost the phosphatase PTEN, which likely plays a role in the strong and consistent activation of Akt phosphorylation observed (Fig. 6A). Furthermore, this level of Akt phosphorylation was shown to be necessary but not sufficient for androgen independent growth, as evidence by the treatment with LY294002. These observations are consistent with reports which have suggested that PI3K is necessary for androgen independent survival, and that MAPK signaling may synergize with PI3K signaling to promote androgen independent growth [27].

In an attempt to confirm the observed increased in Erk phosphorylation in clinical data, immunohistochemistry was performed on a variety of tissue sections from normal prostate tissue through bone metastatic prostate tissue. A significant increase in the level of p-Erk staining was observed in bone metastatic tissue sections as compared to primary tumor and normal prostate tissue, indicating the MAPK activation observed in this *in vitro* model may extend to the clinical setting. This data supports the hypothesis that microenvironment specific, non-oncogene signaling may mediate survival in prostate cancer cells, requiring accurate models of these stromal interactions and assays measuring pathway activation.

In summary, we have established an engineered bone marrow tissue model that exposes prostate cancer cells to various microenvironmental stimuli which orchestrate disease progression, ultimately leading to androgen independent growth. Specifically, using BM-ECM for cell culture studies caused significant alterations in phenotype and cell survival in response to androgen deprivation compared to cells on PTP. Overall these changes appear to more closely mimic *in vivo* disease progression in man. The use of the engineered bone marrow model has potential to accelerate the *in vitro* drug discovery process by overcoming limitations associated with conventional *in vitro* culture studies or the limits with *in vivo* animal models.

## Supporting Information

Figure S1 **Confirmation of MTT correlation to LNCaP cell number.** (A) Standard curves from the same serial dilution of LNCaP cells for MTT and DNA quantification assays. (B) MTT, DNA quantification, and manual count measurements for LNCaP cells on PTP and BM-ECM at three concentrations after 24 h in culture in androgen depleted media (n = 4).
(TIF)

Figure S2 **LNCaP normal growth curve and dose response curves with various inhibitors.** Red dotted lines indicate the doses used in the above experiments. IC50 values were calculated from the dose response curves (n = 12 per concentration).
(TIF)

Figure S3 **Effect of EGF and U0126 on p-Erk phosphorylation.** Western blot confirming the effect of EGF on increasing p-ERK phosphorylation and the effect of U0126 in inhibiting this increased signaling.
(TIF)
