# Supplementary figures and images for: In Vitro Model of Metastasis to Bone Marrow Mediates Prostate Cancer Castration Resistant Growth through Paracrine and Extracellular Matrix Factors
Source: PLoS One. 2012 Aug 1;7(8):e40372. doi: 10.1371/journal.pone.0040372 (PMC3411611; doi:10.1371/journal.pone.0040372)

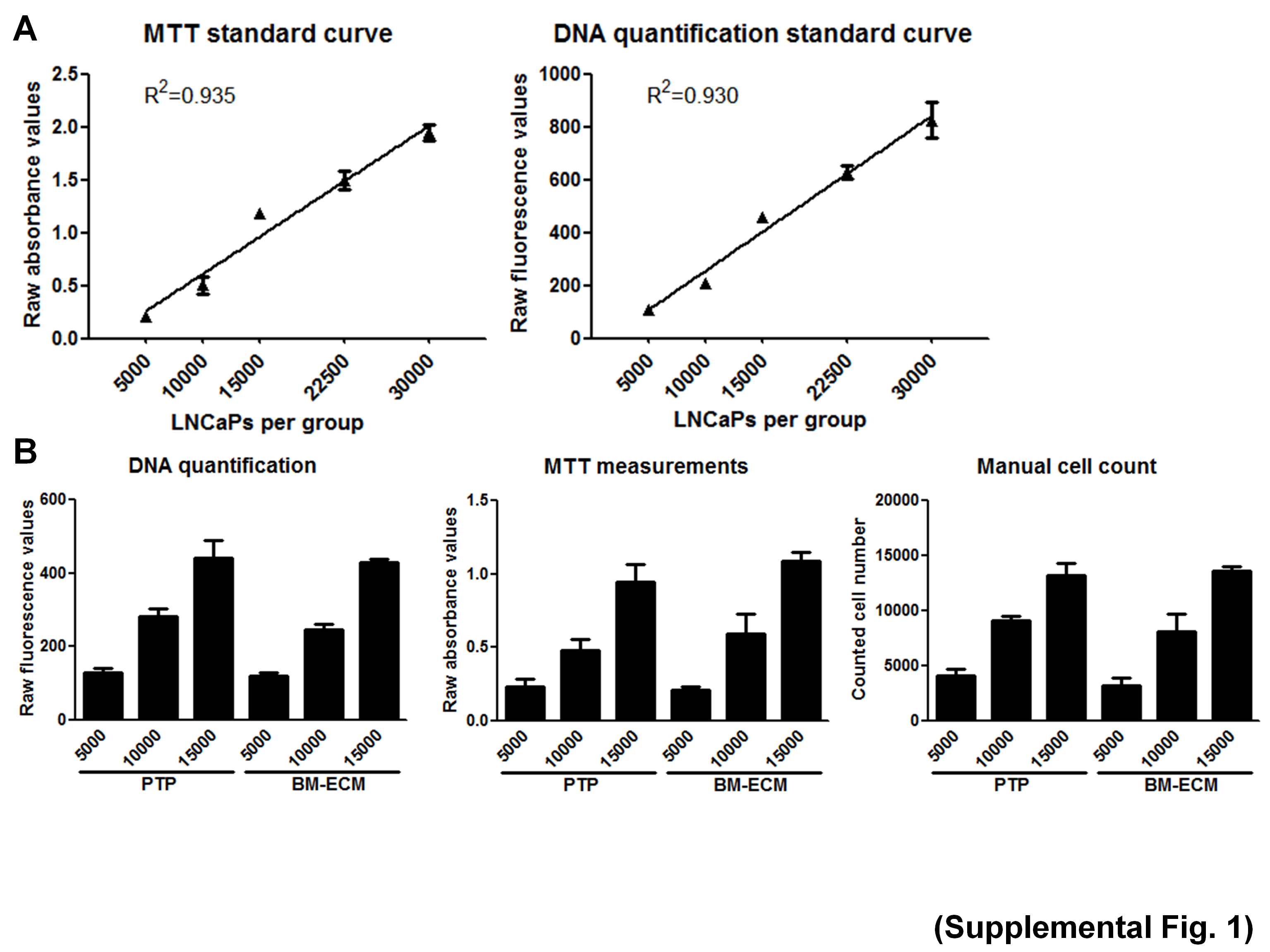

Supplement: Figure S1 — Confirmation of MTT correlation to LNCaP cell number. (A) Standard curves from the same serial dilution of LNCaP cells for MTT and DNA quantification assays. (B) MTT, DNA quantification, and manual count measurements for LNCaP cells on PTP and BM-ECM at three concentrations after 24 h in culture in androgen depleted media (n = 4). (TIF) [file pone.0040372.s001.tif]

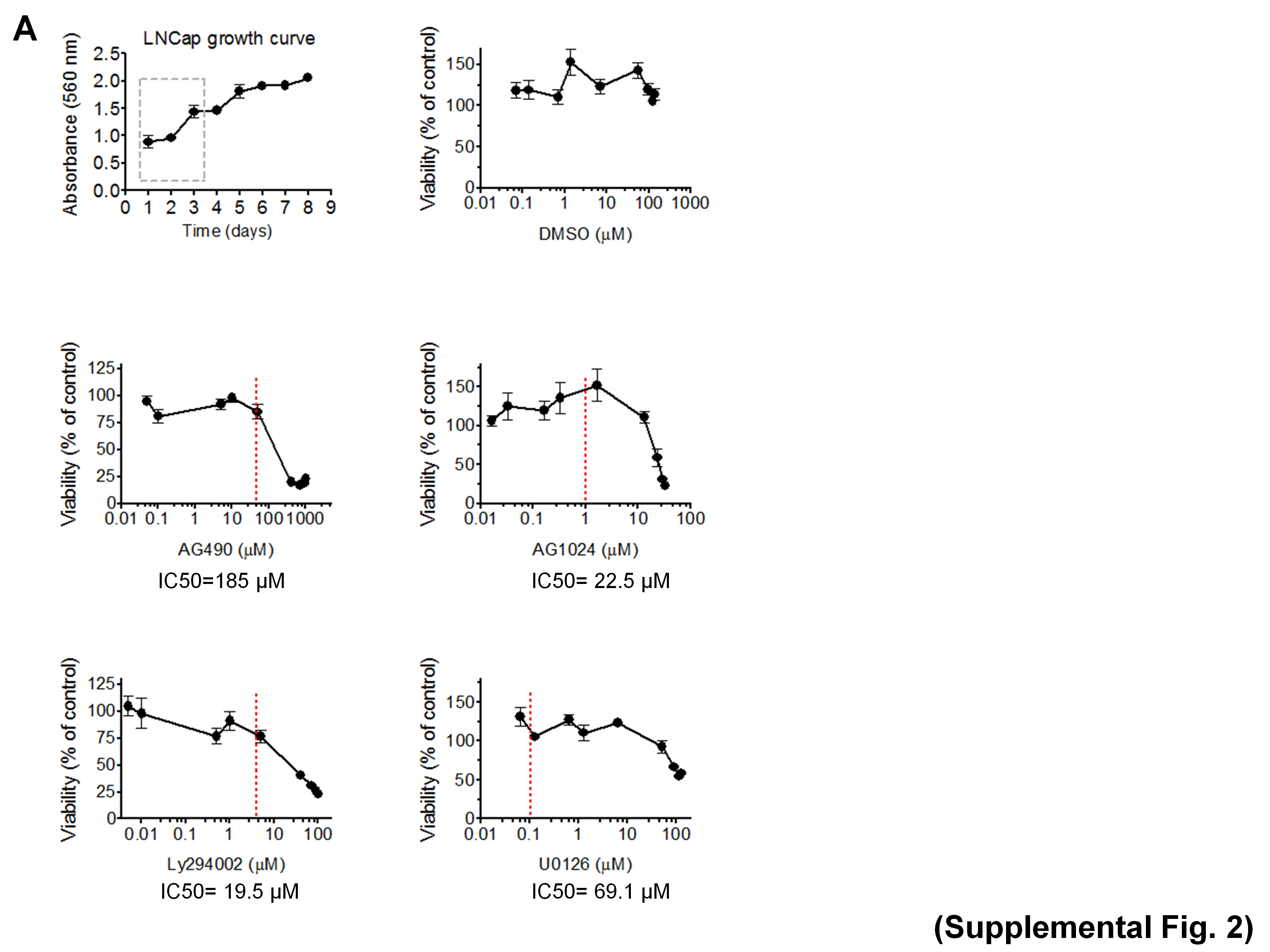

Supplement: Figure S2 — LNCaP normal growth curve and dose response curves with various inhibitors. Red dotted lines indicate the doses used in the above experiments. IC50 values were calculated from the dose response curves (n = 12 per concentration). (TIF) [file pone.0040372.s002.tif]

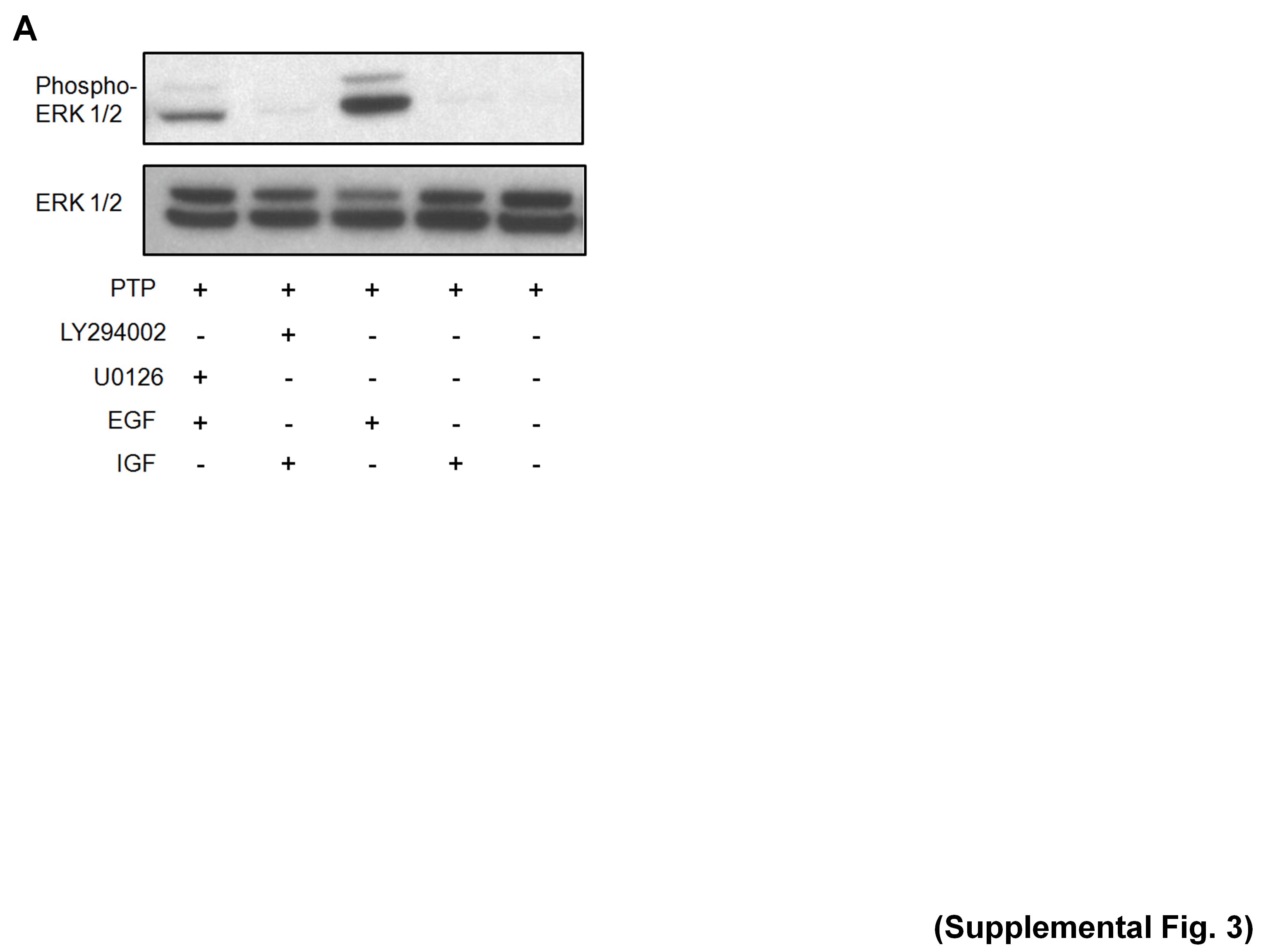

Supplement: Figure S3 — Effect of EGF and U0126 on p-Erk phosphorylation. Western blot confirming the effect of EGF on increasing p-ERK phosphorylation and the effect of U0126 in inhibiting this increased signaling. (TIF) [file pone.0040372.s003.tif]
